# Supplementary material for: A segregated cortical stream for retinal direction selectivity
Source: Nat Commun. 2020 Feb 11;11:831. doi: 10.1038/s41467-020-14643-z (PMC7012930; doi:10.1038/s41467-020-14643-z)
Supplement: Supplementary file 1 — Supplementary Information [file 41467_2020_14643_MOESM1_ESM.pdf]

Supplemental Information

A segregated cortical stream for retinal direction selectivity

Rasmussen et al.

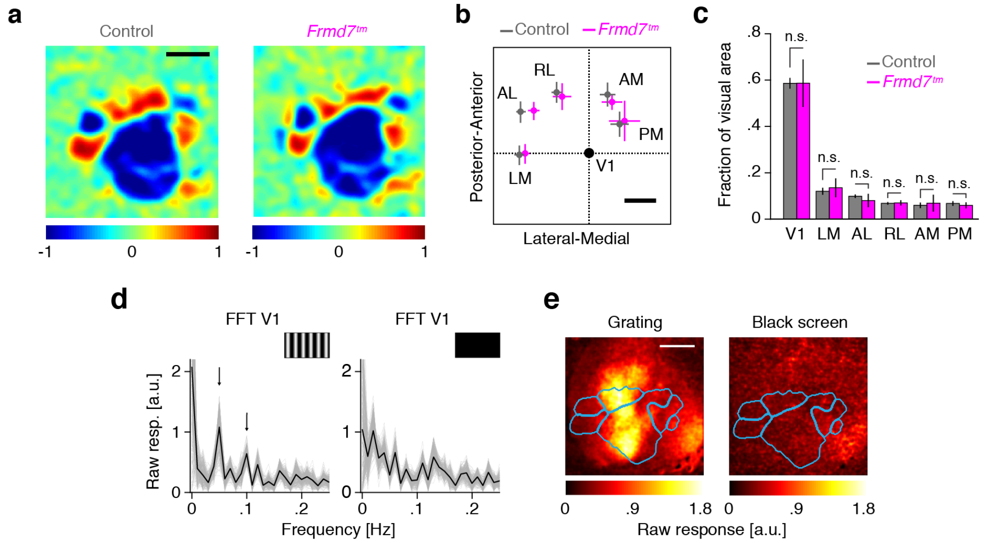

Supplementary Fig. 1: ISOI in control and *Frmd7*^{tm} mice. (a) Example visual field sign maps from control and *Frmd7*^{tm} mice generated using ISOI (scale bar, 1 mm). (b) Higher visual area centers relative to the center of V1 along posterior-anterior and lateral-medial axes (5 mice per group). Error bars are mean ± SEM (scale bar, 0.5 mm). (c) Size of each visual area (5 mice per group) as a fraction of the total (n.s., not significant, two-sided Mann-Whitney U test). Error bars are mean ± SEM. (d) Fast-Fourier transform (FFT) of V1 pixels during grating (left) and black screen (right). Individual pixels are gray, mean is black. (e) Example intrinsic signal response maps depicting the raw response signal for each pixel, determined as the peak power of the visually-evoked signal at the visual stimulus frequency (0.05−0.1 Hz), with visual area borders overlaid. Source data are provided as a Source Data file.

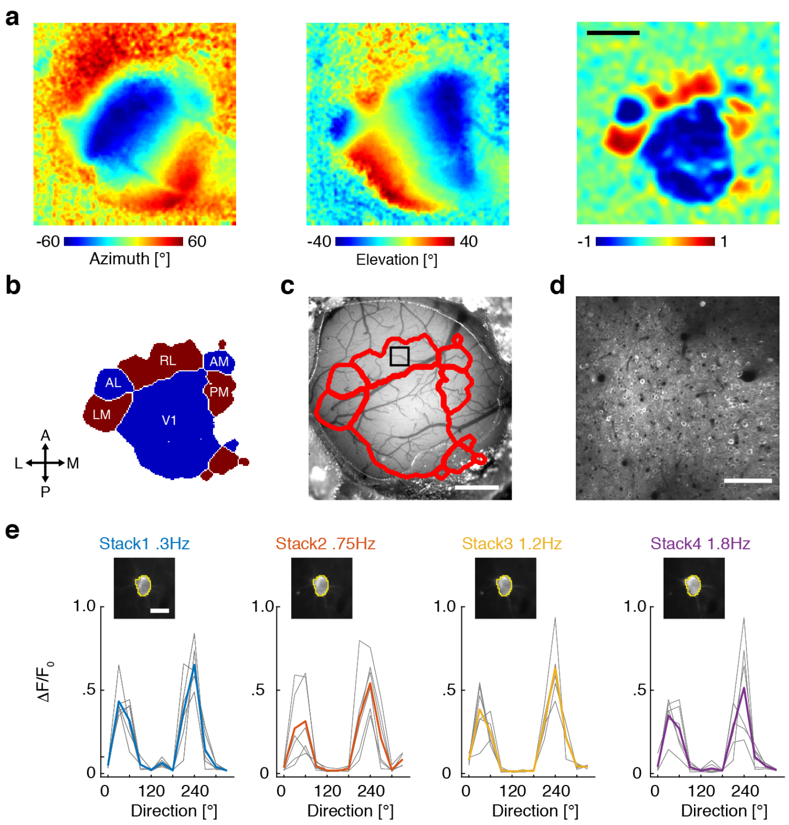

Supplementary Fig. 2: Targeted *in-vivo* two-photon calcium imaging in the visual cortex. (a) Example maps of horizontal (left) and vertical (middle) retinotopy and the corresponding visual field sign (VFS) map from a control mouse (right; scale bar, 1 mm). (b) Thresholded VFS patches computed from the VFS map in (a) showing the location of areas V1, RL and PM, together with the additional three higher visual areas (areas LM, AL, and AM). (c) Visual area borders derived from the VFS patches computed in (b) overlaid on blood vessel map. Black rectangle depicts representative field of view in area RL that was targeted for two-photon calcium imaging (scale bar, 1 mm). (d) Example two-photon mean projection image of RL L2/3 neurons labeled with GCaMP6f in the field of view depicted in (c) (scale bar, 100 µm). (e) Top: Example two-photon mean projection images showing a neuronal somata with overlaid region of interest from the four imaging stacks acquired over a 40 min period (scale bar, 10 µm); one imaging stack per visual temporal frequency condition (0.3, 0.75, 1.2, and 1.8Hz) was acquired. Bottom: Tuning curves at each of the four conditions for the neuron depicted above. Gray lines are individual trials, colored line is the trial-averaged mean.

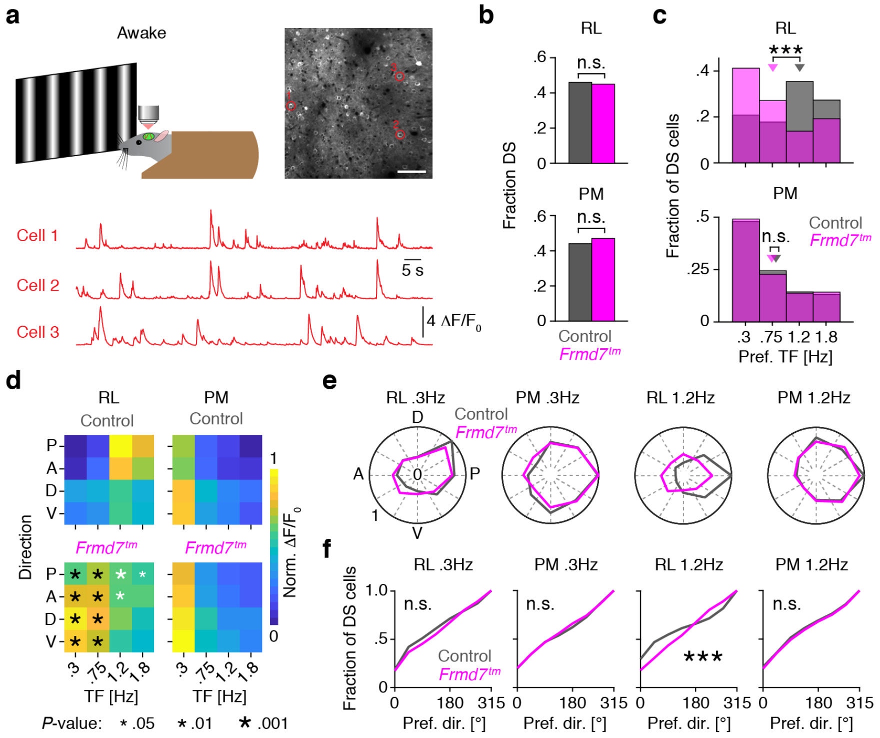

Supplementary Fig. 3: Preference of RL neurons for posterior motion at higher TFs depends on retinal horizontal direction selectivity in awake mice. (a) Two-photon calcium imaging was performed from L2/3 in areas RL and PM of awake control mice (1,652 and 2,018 DS cells, respectively; 4 mice) and *Frmd7*^{tm} mice (2,093 and 4,049 DS cells, respectively; 3 mice). Example image shows two-photon mean projection image of RL neurons expressing GCaMP6f (scale bar, 100 µm). Example traces show activity from three neurons (circled in the image) recorded while the mouse was awake and quietly resting in the cylindrical cover. (b) Fraction of DS cells in RL and PM (two-sided χ^2 test with Yates correction). (c) Preferred TF for DS cells in RL (two-sided Mann-Whitney U test) and PM (two-sided Mann-Whitney U test). Triangles show medians. (d) Response amplitude as a function of motion direction and TF for RL and PM DS cells. White and black asterisks: significantly decreased and increased response amplitude in *Frmd7*^{tm} mice, respectively, two-sided Mann-Whitney U test. (e) Fractional distributions of preferred motion directions for RL and PM DS cells at 0.3 and 1.2Hz; fractions are normalized to the largest fraction across genetic groups. (f) Distributions of preferred direction at 0.3 and 1.2Hz in RL and PM (two-sided Kolmogorov-Smirnov test). **P* < 0.05, ***P* < 0.01, ****P* < 0.001, n.s., not significant, in (b), (c) and (f). Source data are provided as a Source Data file.

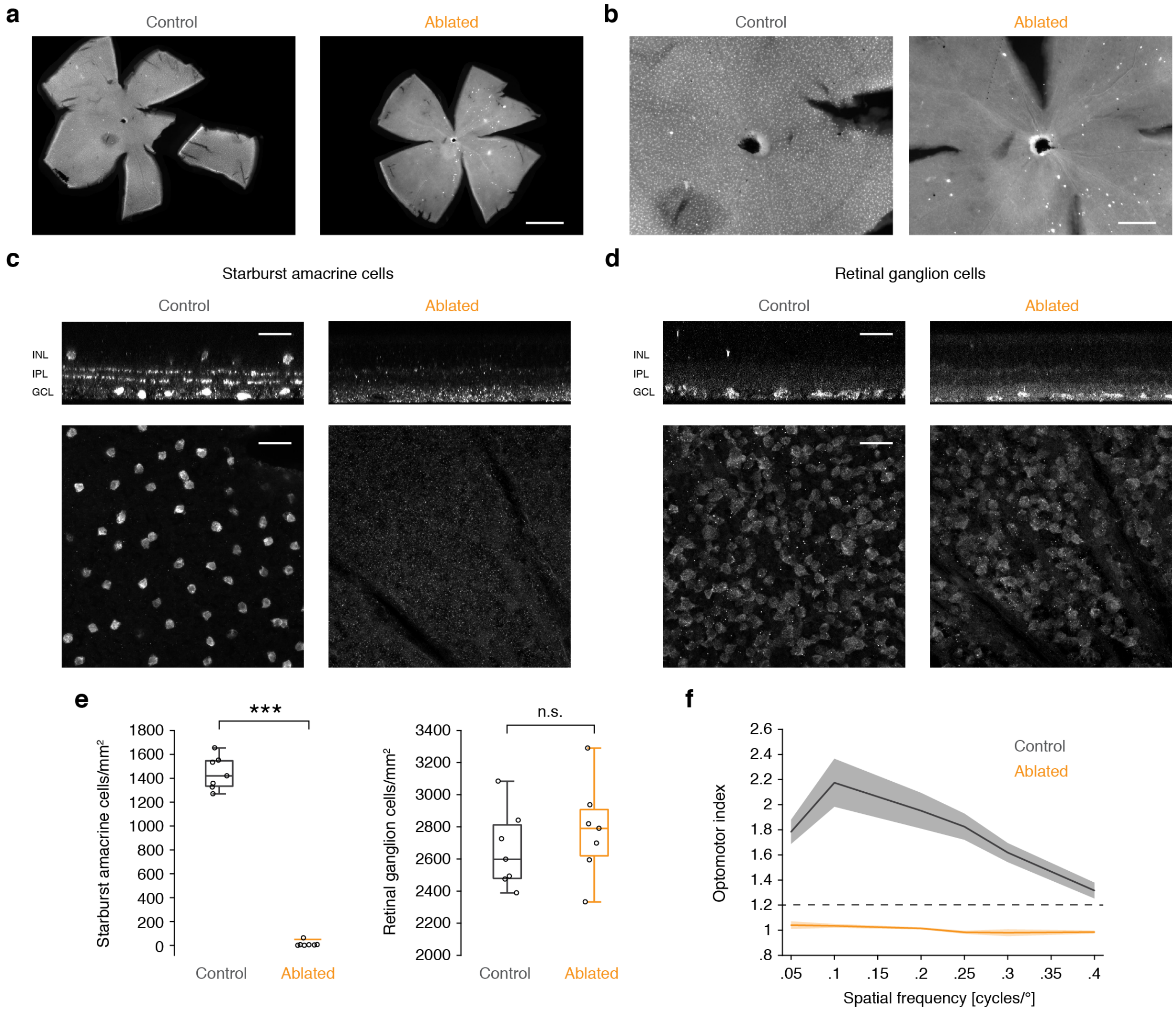

Supplementary Fig. 4: Diphtheria toxin injection selectively ablates starburst amacrine cells in the retina and annihilates optomotor responses. (a) Example whole-mount retinas stained for ChAT to label starburst amacrine cells (white dots show somata) in retinas from control (PBS-injected) and starburst-ablated mice (diphtheria toxin-injected). The white dots in the starburst-ablated retina are fluorescence aggregates, not somata (scale bar, 1 mm). (b) Example higher magnification of the retinas shown in (a) showing the absence of starburst amacrine cells in ablated mice (scale bar, 250 µm). (c) Example side view (top; scale bar, 30 µm) and top view (bottom; scale bar, 30 µm) of retinal z-projection stained for ChAT (starburst amacrine cells) from control (left) and starburst-ablated (right) mice. (d) Example side view (top; scale bar, 30 µm) and top view (bottom; scale bar, 30 µm) of retinal z-projection stained for RBPMS (retinal ganglion cells) from control (left) and starburst-ablated (right) mice. GCL, ganglion cell layer.

INL, inner nuclear layer; IPL, inner plexiform layer. (e) Density quantification of starburst amacrine cells and retinal ganglion cells in control and starburst-ablated mice (7 retinas in each group; $^{***}P < 0.001$, n.s., not significant, two-sided Mann-Whitney U test). Circles are individual data points, center line is median, box limits are 25th and 75th percentiles, and whiskers show minimum and maximum values. (f) Horizontal optomotor response measured in control (5 mice, 7−8 trials per mouse) and starburst-ablated mice (5 mice, 8 trials per mouse). The dotted horizontal line represents the upper quartile of the OMR index previously collected from 3 blind control mice (*rd1*/*rd1* mutants)[1]. Shading indicates SEM.

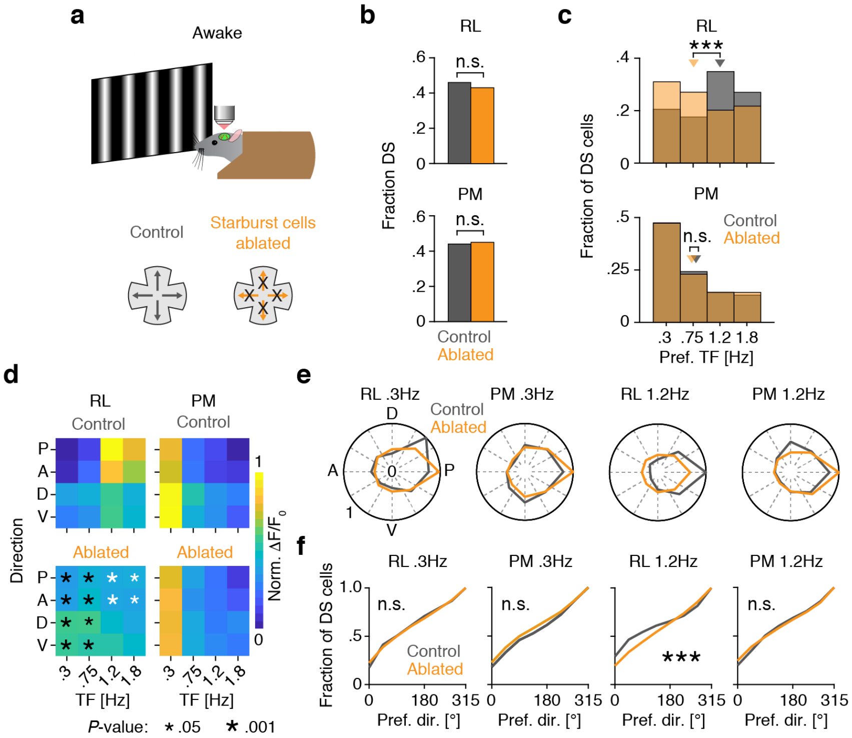

Supplementary Fig. 5: Ablating retinal starburst cells impairs posterior motion preference of RL DS cells at higher TFs. (a) Two-photon calcium imaging was performed from L2/3 in areas RL and PM of awake control mice (1,652 and 2,018 DS cells, respectively; 4 mice) and starburst-cell-ablated mice (2,511 and 2,777 DS cells, respectively; 4 mice). (b) Fraction of DS cells in RL and PM (two-sided χ^2 test with Yates correction). (c) Preferred TF for DS cells in RL (two-sided Mann-Whitney U test) and PM (two-sided Mann-Whitney U test). Triangles show medians. (d) Response amplitude as a function of motion direction and TF for RL and PM DS cells. White and black asterisks: significantly decreased and increased response amplitude in starburst-ablated mice, respectively, two-sided Mann-Whitney U test. (e) Fractional distributions of preferred motion directions for RL and PM DS cells at 0.3 and 1.2Hz; fractions are normalized to the largest fraction across genetic groups. (f) Distributions of preferred direction at 0.3 and 1.2Hz in RL and PM (two-sided Kolmogorov-Smirnov test). ***$P < 0.001$, n.s., not significant, in (b), (c) and (f). Source data are provided as a Source Data file.

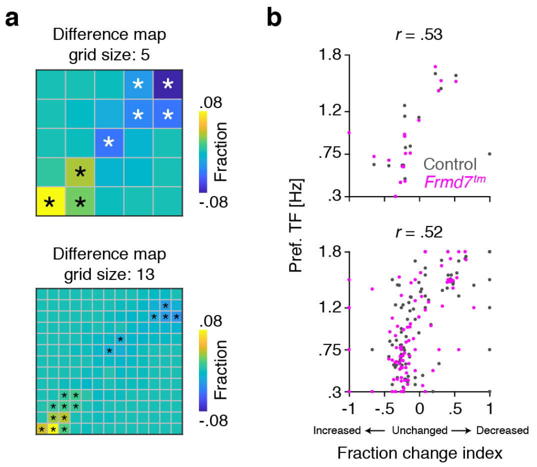

Supplementary Fig. 6: The correlation between the fraction alteration in *Frmd7*^{tm} mice and TF preference is not affected by the grid size used for segmentation. (a) Fraction difference map between control and *Frmd7*^{tm} mice using 5 × 5 (top) or 13 × 13 (bottom) grids in the V1 L2/3 DS cell population (see Fig. 4). Black and white asterisks: significantly decreased and increased fractions in *Frmd7*^{tm} mice, respectively, $P < 0.05$, two-sided χ^2 test with Yates correction. (b) Relationship between fraction changes in *Frmd7*^{tm} mice and the mean preferred TF of each grid for cells from control and *Frmd7*^{tm} mice when 5 × 5 (top) or 13 × 13 (bottom) grids were used. Two-sided Pearson's correlation coefficient is noted on each plot. (c) Two-sided Pearson's correlation coefficient between the fraction change index and mean preferred TF as a function of grid size (top). Corresponding *P*-values testing the significance of the correlation between the fraction change index and mean preferred TF as a function of grid size (bottom).

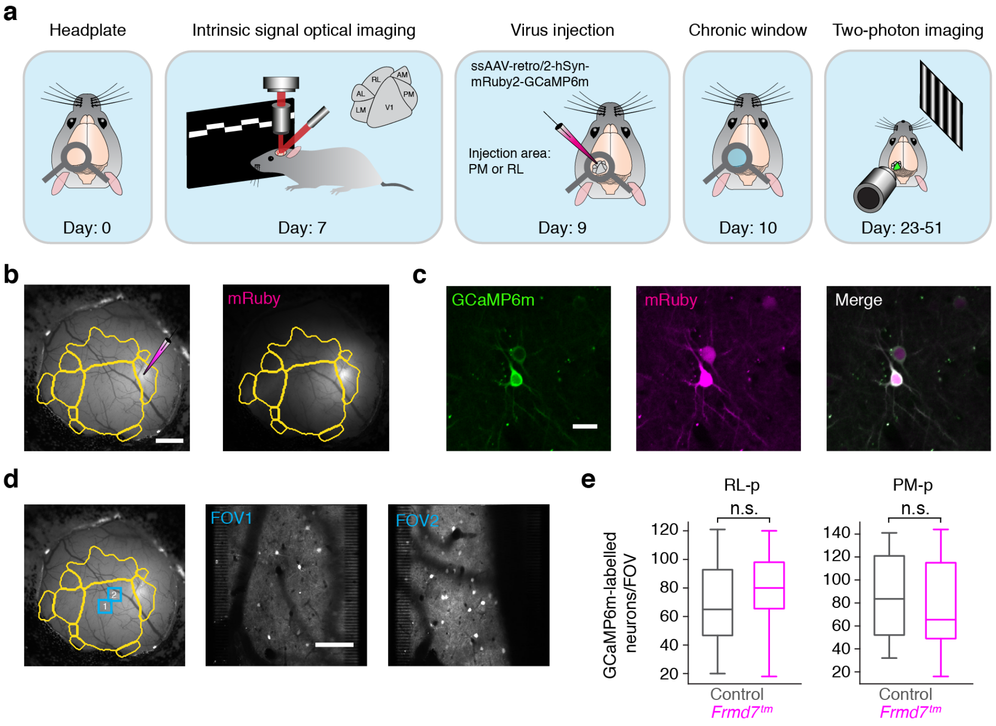

Supplementary Fig. 7: Retrograde viral labeling and GCaMP6 expression in V1 projection neurons. (a) Experimental pipeline employed for achieving GCaMP6 expression in V1 neurons projecting to either area RL or PM. (b) Left: Example image of a cranial window with visual area borders overlaid showing viral (ssAAV-retro/2-hSyn1-mRuby2-GCaMP6m-WPRE) injection site for retrograde expression from area PM in a control mouse (scale bar, 1 mm). Right: mRuby signal in the same cranial window showing site of injection and retrograde expression outside of PM, including in V1. (c) Example V1 neurons expressing GCaMP6m and mRuby (scale bar, 20 µm). (d) Left: Blue rectangles depict fields of view (FOVs) in V1 targeted for two-photon calcium imaging in the same cranial window as shown in (b). Middle and right: Two-photon mean projection images showing neurons labeled with GCaMP6m in the FOVs depicted on the left (scale bar, 100 µm). (e) Number of GCaMP6m-labelled RL-projecting (RL-p) and PM-projecting (PM-p) V1 neurons per FOV in control (21 and 37 FOVs for RL-p and PM-p, respectively) and *Frmd7*^{tm} mice (21 and 34 FOVs for RL-p and PM-p, respectively; n.s., not significant, two-sided Mann-Whitney U test). Center line is median, box limits are 25th and 75th percentiles, and whiskers show minimum and maximum values. Source data are provided as a Source Data file.

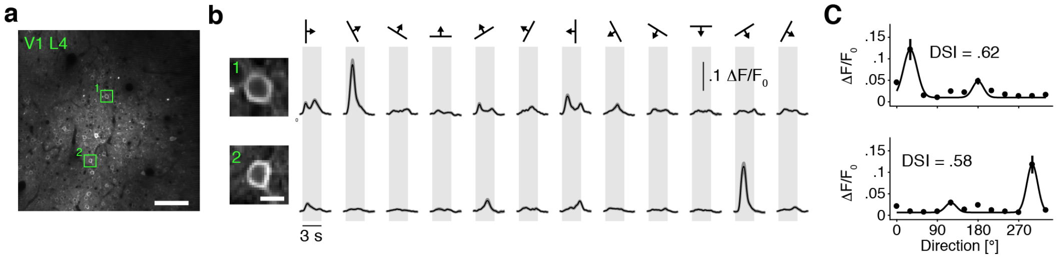

Supplementary Fig. 8: Two-photon calcium imaging from V1 L4 neurons. (a) Example two-photon mean projection image of V1 L4 neurons (395 µm below the dura) labeled with GCaMP6f (scale bar, 100 µm). (b) Example V1 L4 neurons expressing GCaMP6f (scale bar, 10 µm) and trial-averaged fluorescence ($\Delta F/F_0$) time courses for the same neurons. Shading indicates SEM. (c) Tuning curves for the neurons shown in (b). Error bars are SEM; solid line is Gaussian fit.

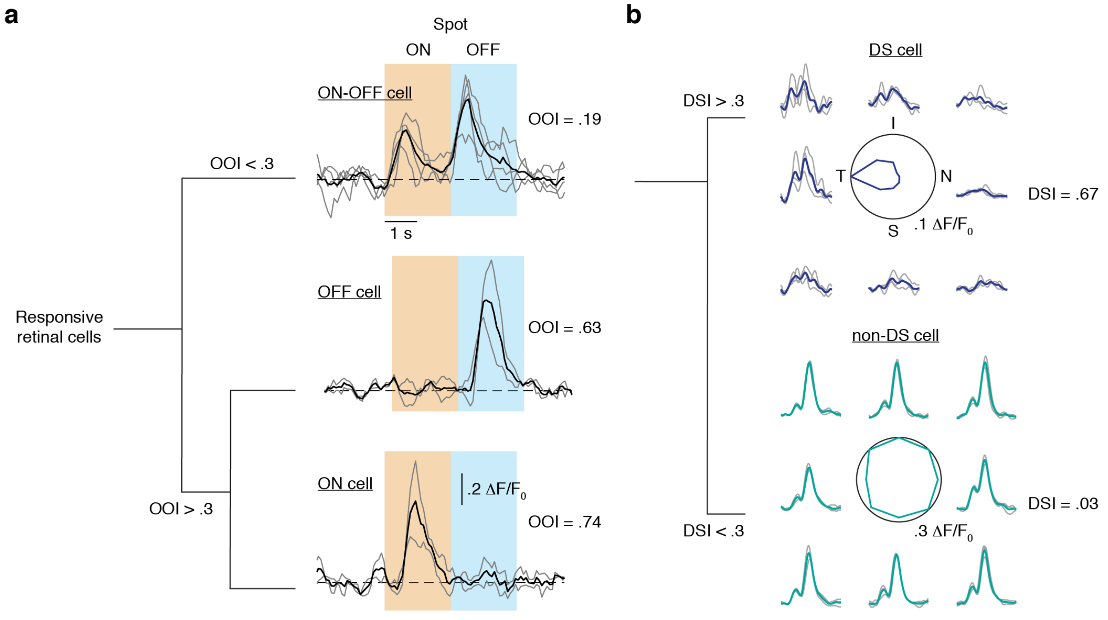

Supplementary Fig. 9: Classification of ON-OFF DS in the retina. (a) To identify ON-OFF DS cells, we first separated the visually responsive retinal cells into two groups: ON-OFF and non-ON-OFF cells, based on an ON-OFF index (OOI, see Methods), denoting the ratio of responses during and after a static flash stimulus (ON phase, orange area; OFF phase, blue area). If cells are responding to both the ON and OFF phase, the OOI value is low: we classified cells with an OOI < 0.3 as ON-OFF cells. Example ON-OFF, OFF, and ON cells are shown. (b) We defined ON-OFF DS cells as ON-OFF cells with a DSI > 0.3. Example ON-OFF DS cells and ON-OFF non-DS cells are shown.

Supplementary references

1. Kretschmer, F., Tariq, M., Chatila, W., Wu, B. & Badea, T. C. Comparison of optomotor and optokinetic reflexes in mice. *J. Neurophysiol.* **118**, 300–316 (2017).
