# Supplementary material for: A segregated cortical stream for retinal direction selectivity
Source: Nat Commun. 2020 Feb 11;11:831. doi: 10.1038/s41467-020-14643-z (PMC7012930; doi:10.1038/s41467-020-14643-z)
Supplement: Supplementary file 3 — Reporting Summary [file 41467_2020_14643_MOESM3_ESM.pdf]

nature research

Corresponding author(s): Keisuke Yonehara

Last updated by author(s): Jan 21, 2020

Reporting Summary

Nature Research wishes to improve the reproducibility of the work that we publish. This form provides structure for consistency and transparency in reporting. For further information on Nature Research policies, see Authors & Referees and the Editorial Policy Checklist .

Statistics

For all statistical analyses, confirm that the following items are present in the figure legend, table legend, main text, or Methods section.

n/a	Confirmed	
☐	☒	The exact sample size (*n*) for each experimental group/condition, given as a discrete number and unit of measurement
☐	☒	A statement on whether measurements were taken from distinct samples or whether the same sample was measured repeatedly
☐	☒	The statistical test(s) used AND whether they are one- or two-sided<br>*Only common tests should be described solely by name; describe more complex techniques in the Methods section.*
☒	☐	A description of all covariates tested
☐	☒	A description of any assumptions or corrections, such as tests of normality and adjustment for multiple comparisons
☐	☒	A full description of the statistical parameters including central tendency (e.g. means) or other basic estimates (e.g. regression coefficient) AND variation (e.g. standard deviation) or associated estimates of uncertainty (e.g. confidence intervals)
☐	☒	For null hypothesis testing, the test statistic (e.g. *F*, *t*, *r*) with confidence intervals, effect sizes, degrees of freedom and *P* value noted<br>*Give P values as exact values whenever suitable.*
☒	☐	For Bayesian analysis, information on the choice of priors and Markov chain Monte Carlo settings
☒	☐	For hierarchical and complex designs, identification of the appropriate level for tests and full reporting of outcomes
☐	☒	Estimates of effect sizes (e.g. Cohen's *d*, Pearson's *r*), indicating how they were calculated

Our web collection on statistics for biologists contains articles on many of the points above.

Software and code

Policy information about availability of computer code

Data collection	LabVIEW version 12.0 (National Instruments), Python 2.7.6 (Python Software Foundation), Psychophysics Toolbox Version 3 (Open Source), SciScan 1.2 (Scientifica) and OKR arena version 0.59.
Data analysis	MATLAB 2017a (MathWorks), Cell Magic Wand Tool (Fitzpatrick Lab), ImageJ v1.52s (NIH) and OKR arena version 0.59.

For manuscripts utilizing custom algorithms or software that are central to the research but not yet described in published literature, software must be made available to editors/reviewers. We strongly encourage code deposition in a community repository (e.g. GitHub). See the Nature Research guidelines for submitting code & software for further information.

Data

Policy information about availability of data

All manuscripts must include a data availability statement. This statement should provide the following information, where applicable:

- Accession codes, unique identifiers, or web links for publicly available datasets
- A list of figures that have associated raw data
- A description of any restrictions on data availability

The data and codes used in this research are available from the corresponding author upon reasonable requests.

Field-specific reporting

Please select the one below that is the best fit for your research. If you are not sure, read the appropriate sections before making your selection.

☒ Life sciences ☐ Behavioural & social sciences ☐ Ecological, evolutionary & environmental sciences

Life sciences study design

All studies must disclose on these points even when the disclosure is negative.

Sample size	Sample size calculation was not performed. The sample sizes were determined as equivalent to previous studies in the field to allow appropriate statistical tests.
Data exclusions	Imaging data were excluded from analysis if motion along the z-axis was detected in order to avoid motion-evoked artifacts contamination the fluorescence data. The data exclusion criteria was predetermined. Otherwise no data were excluded.
Replication	All experiments were replicated in at least 4 mice and often multiple times per mouse (e.g. repeated two-photon imaging over several days for each mouse); all attempts of replication were successful.
Randomization	We did not use any randomization in this study.
Blinding	Data collection and analysis were not performed blind to the conditions of the experiments.

Reporting for specific materials, systems and methods

We require information from authors about some types of materials, experimental systems and methods used in many studies. Here, indicate whether each material, system or method listed is relevant to your study. If you are not sure if a list item applies to your research, read the appropriate section before selecting a response.

Materials & experimental systems

n/a	Involved in the study	
☐	☒	Antibodies
☒	☐	Eukaryotic cell lines
☒	☐	Palaeontology
☐	☒	Animals and other organisms
☒	☐	Human research participants
☒	☐	Clinical data

Methods

n/a	Involved in the study	
☒	☐	ChIP-seq
☒	☐	Flow cytometry
☒	☐	MRI-based neuroimaging

Antibodies

Antibodies used	Primary antibodies: rabbit anti-RBPMS (1:500, Milipore, ABN1362) and goat anti-ChAT (1:200, Milipore, ABN1144P).<br>Secondary antibodies: donkey anti-rabbit IgG Alexa Fluor 568 (1:200, Invitrogen) and donkey anti-goat IgG Alexa Fluor 488 (1:200, Life Technologies).
Validation	RBPMS antibody - PMID: 24318667<br>ChAT antibody - PMID: 29218725; PMID: 26711119; PMID: 21170022<br>anti-rabitt antibody - PMID: 26711119; PMID: 28231464<br>anti-goat antibody - PMID: 26711119; PMID: 24963632

Animals and other organisms

Policy information about studies involving animals; ARRIVE guidelines recommended for reporting animal research

Laboratory animals	Mouse: C57BL/6J (Janvier Labs), Mouse: FRMD7tm1a(KOMP)Wtsi (KOMP Repository), Mouse: FRMD7tm1b(KOMP)Wtsi (KOMP Repository), Mouse: Edil3Tg(Sox2-cre)1Amc/J (Jackson Laboratory), Mouse: Chattm2(cre)Lowl/MwarJ (Jackson laboratory), Mouse: Gt(ROSA)26Sortm1(HBEGF)Awai/J (Jackson Laboratory). All mice were between two and four months old. Mice were group-housed and maintained in a 12-hour/12-hour light/dark cycle with ad libitum access to food and water. Room temperature was between 22 and 24 degrees Celsius and the relative room humidity was maintained between 40 to 60%.
Wild animals	No wild animals were used.
Field-collected samples	No field-collected samples were used.
Ethics oversight	All animal experiments were performed according to standard ethical guidelines and were approved by the Danish National Animal Experiment Committee (Permission No. 2015−15−0201−00541).

Note that full information on the approval of the study protocol must also be provided in the manuscript.
